# Association of a Mediterranean Diet Pattern With Adverse Pregnancy Outcomes Among US Women

**DOI:** 10.1001/jamanetworkopen.2022.48165

**Published:** 2022-12-22

**Authors:** Nour Makarem, Kristi Chau, Eliza C. Miller, Cynthia Gyamfi-Bannerman, Isabella Tous, Whitney Booker, Janet M. Catov, David M. Haas, William A. Grobman, Lisa D. Levine, Rebecca McNeil, C. Noel Bairey Merz, Uma Reddy, Ronald J. Wapner, Melissa S. Wong, Natalie A. Bello

**Affiliations:** 1 Department of Epidemiology, Mailman School of Public Health, Columbia University Irving Medical Center, New York, New York; 2 Department of Neurology, Columbia University Irving Medical Center, New York, New York; 3 Department of Obstetrics, Gynecology and Reproductive Sciences, University of California, San Diego, School of Medicine, San Diego; 4 Vagelos College of Physicians and Surgeons, Columbia University Irving Medical Center, New York, New York; 5 Department of Obstetrics and Gynecology, Columbia University Irving Medical Center, New York, New York; 6 Magee Women’s Research Institute, Department of Obstetrics and Gynecology, University of Pittsburgh School of Medicine, Pittsburgh, Pennsylvania; 7 Department of Obstetrics and Gynecology, Indiana University School of Medicine, Indianapolis; 8 Feinberg School of Medicine, Northwestern University, Chicago, Illinois; 9 Department of Obstetrics and Gynecology, University of Pennsylvania Perelman School of Medicine, Philadelphia; 10 RTI International, Research Triangle Park, North Carolina; 11 Smidt Heart Institute, Cedars-Sinai Medical Center, Los Angeles, California; 12 Department of Obstetrics, Cedars-Sinai Medical Center, Los Angeles, California

## Abstract

**Importance:**

The Mediterranean diet pattern is inversely associated with the leading causes of morbidity and mortality, including metabolic diseases and cardiovascular disease, but there are limited data on its association with adverse pregnancy outcomes (APOs) among US women.

**Objective:**

To evaluate whether concordance to a Mediterranean diet pattern around the time of conception is associated with lower risk of developing any APO and individual APOs.

**Design, Setting, and Participants:**

This prospective, multicenter, cohort study, the Nulliparous Pregnancy Outcomes Study: Monitoring Mothers-to-Be, enrolled 10 038 women between October 1, 2010, and September 30, 2013, with a final analytic sample of 7798 racially, ethnically, and geographically diverse women with singleton pregnancies who had complete diet data. Data analyses were completed between June 3, 2021, and April 7, 2022.

**Exposures:**

An Alternate Mediterranean Diet (aMed) score (range, 0-9; low, 0-3; moderate, 4-5; and high, 6-9) was computed from data on habitual diet in the 3 months around conception, assessed using a semiquantitative food frequency questionnaire.

**Main Outcomes and Measures:**

Adverse pregnancy outcomes were prospectively ascertained and defined as developing 1 or more of the following: preeclampsia or eclampsia, gestational hypertension, gestational diabetes, preterm birth, delivery of a small-for-gestational-age infant, or stillbirth.

**Results:**

Of 7798 participants (mean [SD] age, 27.4 [5.5] years), 754 (9.7%) were aged 35 years or older, 816 (10.5%) were non-Hispanic Black, 1294 (16.6%) were Hispanic, and 1522 (19.5%) had obesity at baseline. The mean (SD) aMed score was 4.3 (2.1), and the prevalence of high, moderate, and low concordance to a Mediterranean diet pattern around the time of conception was 30.6% (n=2388), 31.2% (n=2430), and 38.2% (n=2980), respectively. In multivariable models, a high vs low aMed score was associated with 21% lower odds of any APO (adjusted odds ratio [aOR], 0.79 [95% CI, 0.68-0.92]), 28% lower odds of preeclampsia or eclampsia (aOR, 0.72 [95% CI, 0.55-0.93]), and 37% lower odds of gestational diabetes (aOR, 0.63 [95% CI, 0.44-0.90]). There were no differences by race, ethnicity, and prepregnancy body mass index, but associations were stronger among women aged 35 years or older (aOR, 0.54 [95% CI, 0.34-0.84]; *P* = .02 for interaction). When aMed score quintiles were evaluated, similar associations were observed, with higher scores being inversely associated with the incidence of any APO.

**Conclusions and Relevance:**

This cohort study suggests that greater adherence to a Mediterranean diet pattern is associated with lower risk of APOs, with evidence of a dose-response association. Intervention studies are needed to assess whether dietary modification around the time of conception can reduce risk of APOs and their downstream associations with future development of cardiovascular disease risk factors and overt disease.

## Key Points

**Question:**

Among geographically, racially, and ethnically diverse nulliparous US women, is concordance to a Mediterranean diet around the time of conception associated with risk of developing any adverse pregnancy outcome (APO) and individual APOs?

**Findings:**

In this cohort study of 7798 women, greater concordance to a Mediterranean diet pattern was significantly associated with 21% lower risk of developing any APO, with evidence of a dose-response association. There were no differences by race, ethnicity, and prepregnancy body mass index, but associations were stronger among older women.

**Meaning:**

This study suggests that the Mediterranean diet pattern is inversely associated with APOs; intervention studies are needed to assess whether promoting a Mediterranean-style diet around the time of conception and throughout pregnancy can prevent APOs.

## Teaser

This cohort study evaluates whether concordance to a Mediterranean diet pattern around the time of conception is associated with lower risk of developing any adverse pregnancy outcome and individual adverse pregnancy outcomes.

## Introduction

A Centers for Disease Control and Prevention’s 2022 National Center for Health Statistics report indicates that pregnancy-related mortality in the United States has been on the increase steadily over the past 30 years, with significant disparities by race and maternal age.^1^ Adverse pregnancy outcomes (APOs) are leading factors associated with maternal morbidity and mortality, underscoring the importance of APO prevention for preserving and extending a healthy lifespan among women.^2^ APOs have been associated with an increased risk of the subsequent development of metabolic diseases, cardiovascular disease (CVD) risk factors, and overt CVD.^2^ As such, a history of APO is considered a risk enhancer and a prompt for more vigorous lifestyle interventions for primary prevention of CVD, the leading cause of death among US women.^2^

Prior work has shown a high prevalence of poor diet quality among US women periconceptionally,^3^ and little change of dietary patterns from before pregnancy to early pregnancy.^4^ Thus, a woman’s periconceptual diet may be reflective of general nutritional habits and future diet, and represents an important potential target for reducing APOs and extending a healthy lifespan.^5^ The Mediterranean diet pattern, which has been linked to health and longevity, is characterized by high intake of plant-based foods, such as vegetables, legumes, fruits, nuts, and monounsaturated fats, coupled with a low intake of saturated fats and processed meats.^6,7,8,9,10^ Greater adherence to a Mediterranean diet pattern has been associated with a lower risk for multiple chronic diseases and mortality^6,7,8,9,10,11,12^; we hypothesized that it was associated with a reduced risk of APOs. Only 3 observational studies with modest sample sizes have previously investigated the association of adherence to this diet pattern around the time of conception with risk of developing APOs.^13,14,15^ Two studies focused on gestational diabetes^13,14^ and only 1 examined preeclampsia as an outcome.^15^ Furthermore, the role of social determinants of health, previously linked to APOs and known to influence choice in dietary characteristics and diet quality, in these associations has not been fully elucidated.^2,16,17,18,19^

To address this knowledge gap, we evaluated the association of an Alternate Mediterranean Diet (aMed) score, which is comprised of foods that are characteristic of the Mediterranean pattern but adapted for the US population,^7^ and its components with odds of developing any APO and individual APOs using data from the ongoing, prospective Nulliparous Pregnancy Outcomes Study: Monitoring Mothers-to-Be (nuMoM2b), one of the largest population-based cohort studies of US pregnant women.^20^

## Methods

### Study Population

Full details of the nuMoM2b study have been described elsewhere.^20^ This multicenter cohort study was conducted at 8 US medical centers from October 1, 2010, to September 30, 2013, and enrolled 10 038 nulliparous women with live singleton pregnancies in their first trimester and followed them through delivery. Each study site’s local institutional review board (Case Western Reserve University, Cleveland, Ohio; Columbia University, New York, New York; Indiana University, Indianapolis; University of Pittsburgh, Pittsburgh, Pennsylvania; Northwestern University, Chicago, Illinois; University of California at Irvine; University of Pennsylvania, Philadelphia; and University of Utah, Salt Lake City) approved the study protocol, and all women provided written informed consent. At the first study visit, extensive sociodemographic, lifestyle, and medical data were collected. Women were excluded from the present analysis due to incompleteness of diet data, implausible energy intakes, or history of chronic hypertension or diabetes, resulting in an analytic sample of n = 7798 (eFigure in Supplement 1). This study followed the reporting requirements of the Strengthening the Reporting of Observational Studies in Epidemiology (STROBE) statement.^21^

### Assessment of Habitual Diet

Diet around the time of conception was assessed using the modified Block 2005 Food Frequency Questionnaire (FFQ) at the first study visit between 6 weeks and 13 weeks plus 6 days of gestation.^3^ This semiquantitative FFQ evaluated habitual dietary intake during the past 3 months (ie, periconceptionally) by querying participants about the amount and frequency of consumption of approximately 120 food and beverage items to assess intakes of 52 nutrients and 35 food groups. The FFQ, which was administered in English or Spanish, has been validated in pregnant women.^22,23,24,25^ It was slightly modified from the original version to inquire about usual diet over the past 3 months and include additional sources of ω3 fatty acids.^3^ All FFQs were checked by study staff for completeness and were sent for analysis by Block Dietary Data Systems.

### Operationalization of the aMed Score

Concordance to a Mediterranean diet pattern was evaluated by computing an aMed score using data on habitual periconceptual diet from the Block FFQ.^22,23^ All diet variables were energy adjusted using the nutrient density method.^26^ We used the approach described by Fung et al,^7^ which captures adherence to this diet pattern, while adapting the original Mediterranean diet scale described by Trichopoulou et al^6^ for US populations. The aMed score consists of 9 components: vegetables (excluding potatoes), fruits, nuts, whole grains, legumes, fish, monounsaturated to saturated fat ratio, red and processed meats, and alcohol. Participants received a score for each component, such that those with intake above the median for vegetables, fruits, nuts, whole grains, legumes, fish, and monounsaturated to saturated fat ratio received a score of 1; otherwise, they received a score of 0. For red and processed meat consumption, those with intakes below the median were assigned a score of 1 and those with intakes above the median were assigned a score of 0. For alcohol intake, women who consumed between 5 and 15 g/d, representing approximately one 12-oz can of beer, 5 oz of wine, or 1.5 oz of liquor, received a score of 1; otherwise, they received a score of 0. The component scores were then summed to create the overall aMed score, which ranged from 0 to 9, with a higher score representing closer resemblance to the Mediterranean diet.

### Ascertainment of APOs

The primary outcome was the development of any APO, defined as developing 1 or more of the following: gestational hypertension, preeclampsia or eclampsia,^27^ gestational diabetes, preterm birth (medically indicated or spontaneous live birth at <37 weeks’ gestational age; assessed as both a composite and as spontaneous or iatrogenic preterm birth), delivery of a small-for-gestational-age infant (<5th percentile by Alexander nomogram),^28^ or stillbirth. In secondary analyses, we examined the individual APOs. All outcomes were adjudicated by a panel of maternal-fetal medicine experts.

### Statistical Analysis

Data analyses were completed between June 3, 2021, and April 7, 2022. Sociodemographic and clinical characteristics were described as mean (SD) values for continuous variables and frequencies for categorical variables. We used χ^2^ and analysis of variance tests as appropriate to evaluate whether these characteristics were statistically significantly different across predefined categories of the aMed score indicative of low, moderate, and high adherence to this diet pattern using score cutoffs consistent with prior research (low, 0-3; moderate, 4-5; high, 6-9).^8,10^ Univariable and multivariable logistic regression models evaluated the aMed score and its component scores in association with the odds of developing any APO (primary outcome) and individual APOs (secondary outcomes). Multivariable models were adjusted for a priori defined potential confounders including maternal age (years), marital status (married vs single, divorced, separated, or widowed), educational level (college education or greater vs no college), self-reported race and ethnicity (Asian, Hispanic, non-Hispanic Black, non-Hispanic White, and other [self-reported categories of American Indian, Native Hawaiian, multiracial, and other]), smoking (ever vs never), body mass index (BMI; calculated as weight in kilograms divided by height in meters squared) category (≥30 vs <30), and family history of CVD (yes vs no). Two analytic approaches were used: (1) using predefined aMed score categories (low, moderate, high)^8,10^ with low aMed score as the reference group and (2) a data-driven approach where quintiles of the aMed score (quintile 1: aMed score, 0-2; quintile 2: aMed score, 3-4; quintile 3: aMed score, 5; quintile 4: aMed score, 6; quintile 5: aMed score, 7-9) were examined, with quintile 1 as the reference group.^7,11^ A test for linear trend across quintiles of aMed score was performed to detect whether there was a dose-response association between aMed adherence and APOs. In sensitivity analyses, we examined whether additional adjustment for percentage of the federal poverty level and health insurance altered our primary analyses evaluating aMed score categories in association with the composite outcome (any APO).

In prespecified exploratory analyses, we tested for interactions in the associations of aMed score categories with APOs by self-reported race (non-Hispanic Black vs non-Hispanic White), ethnicity (Hispanic vs Non-Hispanic White), prepregnancy obesity (BMI category ≥30 vs <30), and maternal age (≥35 years vs <35 years). If a statistically significant interaction was detected (*P* < .05 for interaction), subgroup analyses were conducted. All statistical tests were 2-tailed, and *P* < .05 was considered significant. Statistical analyses were performed using R, version 4.1.0 (R Project for Statistical Computing),^29^ and Stata/MP, version 15 (StataCorp LLC), was used for computation of *P* values for trends.

## Results

Characteristics of the 7798 included participants are displayed in Table 1. The mean (SD) age was 27.4 (5.5) years and 754 women (9.7%) were aged 35 years or older. The racial and ethnic distribution was 4.3% Asian (n = 337), 16.6% Hispanic (n = 1294), 10.5% non-Hispanic Black (n = 816), and 63.9% non-Hispanic White (n = 4986). About half the women (3718 [47.7%]) had an educational level equivalent to a Bachelor’s degree and above, 5029 (64.5%) were married, and 1522 (19.5%) had obesity. The mean (SD) aMed score was 4.3 (2.1). Overall, the prevalence of high, moderate, and low concordance to a Mediterranean diet pattern around the time of conception was 30.6% (n = 2388), 31.2% (n = 2430), and 38.2% (n = 2980), respectively. When sociodemographic and clinical characteristics were compared across predefined categories of the aMed score, women with a higher aMed score were more likely to be older (mean [SD], 30.1 [4.4] years; *P* < .001), non-Hispanic White (1855 of 2388 [77.7%]; *P* < .001), married (2075 of 2388 [86.9%]; *P* < .001), never smokers (1424 of 2388 [59.6%]; *P* < .001), and have a higher educational level (1671 of 2388 [70.0%]; *P* < .001) and less likely to have a BMI in the obesity category (300 of 2388 [12.6%]; *P* < .001). Participants in the high vs low aMed score category had lower overall prevalence of any APO (761 of 2388 [31.9%] vs 1137 of 2980 [38.2%]; *P* < .001) (eTable 1 in Supplement 1), including a significantly lower prevalence of preeclampsia (146 of 2388 [6.1%] vs 276 of 2980 [9.3%]; *P* < .001) and delivery of a small-for-gestational-age infant (209 of 2388 [8.8%] vs 331 of 2980 [11.1%]; *P* < .001).

**Table 1.** Participant Characteristics at the Time of Pregnancy

Characteristic	Overall, No. (%) (N = 7798)	aMed dietary adherence, No. (%)^a^			*P* value
		Low (n = 2980)	Moderate (n = 2430)	High (n = 2388)	
Age, mean (SD), y	27.4 (5.5)	24.6 (5.3)	28.0 (5.3)	30.1 (4.4)	<.001
Advanced maternal age (≥35 y)	754 (9.7)	146 (4.9)	269 (11.1)	339 (14.2)	<.001
Race and ethnicity					
Asian	337 (4.3)	66 (2.2)	145 (6.0)	126 (5.3)	<.001
Hispanic	1294 (16.6)	641 (21.5)	415 (17.1)	238 (10.0)	
Non-Hispanic Black	816 (10.5)	586 (19.7)	166 (6.8)	64 (2.7)	
Non-Hispanic White	4986 (63.9)	1522 (51.1)	1609 (66.2)	1855 (77.7)	
Other^b^	362 (4.6)	163 (5.5)	94 (3.9)	105 (4.4)	
Educational level					
<High school, no diploma	384 (4.9)	296 (9.9)	78 (3.2)	10 (0.4)	<.001
High school graduate or GED	838 (10.7)	594 (19.9)	198 (8.1)	46 (1.9)	
<College degree	1753 (22.5)	959 (32.2)	505 (20.8)	289 (12.1)	
≥Bachelor’s degree	3718 (47.7)	767 (25.7)	1280 (52.7)	1671 (70.0)	
Marital status					
Single	2681 (34.4)	1717 (57.6)	666 (27.4)	298 (12.5)	<.001
Married	5029 (64.5)	1224 (41.1)	1730 (71.2)	2075 (86.9)	
Other (divorced, separated, widowed)	83 (1.1)	37 (1.2)	31 (1.3)	15 (0.6)	
BMI in early pregnancy, mean (SD)	25.9 (6.0)	26.7 (6.7)	26.1 (5.9)	24.8 (4.7)	<.001
Obesity (BMI ≥30)	1522 (19.5)	730 (24.5)	492 (20.2)	300 (12.6)	<.001
Ever smoker	3231 (41.4)	1335 (44.8)	932 (38.4)	964 (40.4)	<.001
Family history of cardiovascular disease	579 (7.4)	169 (5.7)	199 (8.2)	211 (8.8)	<.001

Abbreviations: aMed, Alternate Mediterranean Diet; BMI, body mass index (calculated as weight in kilograms divided by height in meters squared); GED, General Educational Development certification.

^a^ aMed score categories defined as low, 0 to 3; moderate, 4 to 5; and high, 6 to 9.

^b^ Includes self-reported categories of American Indian, Native Hawaiian, multiracial, and other.

### Associations of Predefined aMed Score Categories With APOs

In multivariable models, participants with a high vs low aMed score had 21% lower odds of any APO (adjusted odds ratio [aOR], 0.79 [95% CI, 0.68-0.92]) (Table 2). A high vs low aMed score was also associated with 28% lower odds of preeclampsia or eclampsia (aOR, 0.72 [95% CI, 0.55-0.93]) and 37% lower odds of gestational diabetes (aOR, 0.63 [95% CI, 0.44-0.90]). The aMed score was not significantly associated with odds of developing gestational hypertension, preterm birth, delivering a small-for-gestational-age infant, or stillbirth.

**Table 2.** Univariate and Multivariable Associations of Predefined aMed Score Categories With Odds of Having Any APO and Individual APOs^a^

Diet score adherence	Unadjusted		Adjusted^b^	
	OR (95% CI)	*P* value	OR (95% CI)	*P* value
Any APO (n = 2747)				
Low aMed score	1 [Reference]	NA	1 [Reference]	NA
Moderate aMed score	0.88 (0.78-0.98)	.02	0.90 (0.79-1.03)	.14
High aMed score	0.76 (0.67-0.85)	<.001	0.79 (0.68-0.92)	.002
Preeclampsia or eclampsia (n = 606)				
Low aMed score	1 [Reference]	NA	1 [Reference]	NA
Moderate aMed score	0.80 (0.66-0.97)	.03	0.85 (0.68-1.07)	.17
High aMed score	0.64 (0.52-0.78)	<.001	0.72 (0.55-0.93)	.01
Gestational hypertension (n = 1106)				
Low aMed score	1 [Reference]	NA	1 [Reference]	NA
Moderate aMed score	0.92 (0.78-1.07)	.26	0.89 (0.74-1.07)	.21
High aMed score	0.92 (0.79-1.07)	.27	0.85 (0.70-1.03)	.11
Gestational diabetes (n = 300)				
Low aMed score	1 [Reference]	NA	1 [Reference]	NA
Moderate aMed score	1.05 (0.80-1.37)	.71	0.88 (0.64-1.20)	.41
High aMed score	0.75 (0.56-1.00)	.05	0.63 (0.44-0.90)	.01
Preterm birth–composite (n = 544)				
Low aMed score	1 [Reference]	NA	1 [Reference]	NA
Moderate aMed score	0.89 (0.72-1.09)	.25	0.95 (0.74-1.21)	.69
High aMed score	0.79 (0.64-0.98)	.03	0.91 (0.69-1.20)	.52
Preterm birth–iatrogenic (n = 181)				
Low aMed score	1 [Reference]	NA	1 [Reference]	NA
Moderate aMed score	0.89 (0.63-1.25)	.49	0.98 (0.66-1.46)	.92
High aMed score	0.66 (0.45-0.96)	.03	0.80 (0.49-1.27)	.34
Preterm birth–spontaneous (n = 363)				
Low aMed score	1 [Reference]	NA	1 [Reference]	NA
Moderate aMed score	0.89 (0.69-1.15)	.38	0.94 (0.69-1.26)	.67
High aMed score	0.87 (0.68-1.13)	.30	0.98 (0.70-1.37)	.91
Small size for gestational age (n = 765)				
Low aMed score	1 [Reference]	NA	1 [Reference]	NA
Moderate aMed score	0.82 (0.68-0.98)	.03	1.00 (0.81-1.23)	.99
High aMed score	0.77 (0.64-0.92)	.005	1.03 (0.81-1.31)	.79
Stillbirth (n = 38)				
Low aMed score	1 [Reference]	NA	1 [Reference]	NA
Moderate aMed score	0.51 (0.21-1.14)	.12	0.63 (0.23-1.56)	.34
High aMed score	0.72 (0.33-1.49)	.39	1.02 (0.39-2.61)	.97

Abbreviations: aMed, Alternate Mediterranean Diet; APO, adverse pregnancy outcome; NA, not applicable; OR, odds ratio.

^a^ Logistic regression models were used to evaluate associations of aMed categories (low, 0-3; moderate, 4-5; and high, 6-9) with odds of having any and individual APOs.

^b^ Model is adjusted for age, educational level, race and ethnicity, marital status, obesity, smoking, and family history of cardiovascular disease.

### Associations of aMed Score Quintiles With APOs

Participants in the highest vs lowest quintile of the aMed score had 20% lower odds of having any APO (aOR, 0.80 [95% CI, 0.66-0.97]), and a statistically significant linear trend indicative of a dose-response association was detected across quintiles of the aMed score (second quintile: aOR, 1.01 [95% CI, 0.87-1.17]; third quintile: aOR, 0.83 [95% CI, 0.69-1.00]; fourth quintile: aOR, 0.79 [95% CI, 0.65-0.96]; fifth quintile: aOR, 0.80 [95% CI, 0.66-0.97]; *P* = .007 for trend) (Table 3). This finding was unchanged when additional adjustment for percentage of the federal poverty level and health insurance was added to the multivariable model in sensitivity analyses (OR, 0.79 [95% CI, 0.66-0.94]). In analyses evaluating associations of the aMed score with individual APOs, those in the highest vs lowest quintile had 35% (95% CI, 8%-54%) lower odds of any preeclampsia or eclampsia and 54% (95% CI, 25%-72%) lower odds of gestational diabetes.

**Table 3.** Univariate and Multivariable Associations of aMed Score Quintiles With Odds of Having Any APO and Individual APOs^a^

Diet score adherence	Unadjusted			Adjusted^b^		
	OR (95% CI)	*P* value	*P* value for trend	OR (95% CI)	*P* value	*P* value for trend
Any APO (n = 2747)						
Quintile^c^						
First	1 [Reference]	NA		1 [Reference]	NA	
Second	0.92 (0.81-1.04)	.19	<.001	1.01 (0.87-1.17)	.92	.007
Third	0.74 (0.64-0.87)	<.001		0.83 (0.69-1.00)	.05	
Fourth	0.72 (0.61-0.84)	<.001		0.79 (0.65-0.96)	.02	
Fifth	0.73 (0.62-0.85)	<.001		0.80 (0.66-0.97)	.02	
Preeclampsia or eclampsia (n = 606)						
Quintile^c^						
First	1 [Reference]	NA		1 [Reference]	NA	
Second	0.92 (0.75-1.13)	.43	<.001	0.93 (0.73-1.18)	.54	.01
Third	0.58 (0.43-0.77)	<.001		0.61 (0.44-0.86)	.005	
Fourth	0.61 (0.45-0.81)	<.001		0.67 (0.48-0.94)	.02	
Fifth	0.61 (0.46-0.80)	<.001		0.65 (0.46-0.92)	.02	
Gestational hypertension (n = 1106)						
Quintile^c^						
First	1 [Reference]	NA		1 [Reference]	NA	
Second	0.92 (0.78-1.10)	.37	.77	0.98 (0.81-1.19)	.82	.77
Third	0.89 (0.72-1.09)	.26		0.90 (0.70-1.15)	.41	
Fourth	0.88 (0.71-1.09)	.25		0.85 (0.66-1.10)	.23	
Fifth	0.91 (0.74-1.12)	.38		0.89 (0.69-1.15)	.36	
Gestational diabetes (n = 300)						
Quintile^c^						
First	1 [Reference]	NA		1 [Reference]	NA	
Second	0.86 (0.63-1.16)	.31	.14	0.72 (0.51-1.02)	.06	.07
Third	0.86 (0.59-1.23)	.40		0.73 (0.48-1.12)	.15	
Fourth	0.72 (0.48-1.06)	.10		0.61 (0.38-0.95)	.03	
Fifth	0.61 (0.40-0.90)	.01		0.46 (0.28-0.75)	.002	
Preterm birth–composite (n = 544)						
Quintile^c^						
First	1 [Reference]	NA		1 [Reference]	NA	
Second	0.88 (0.70-1.11)	.28	.10	1.04 (0.80-1.34)	.78	.66
Third	0.78 (0.58-1.03)	.08		0.92 (0.65-1.30)	.65	
Fourth	0.69 (0.51-0.93)	.02		0.81 (0.55-1.17)	.26	
Fifth	0.80 (0.60-1.06)	.12		1.06 (0.74-1.51)	.74	
Small for gestational age (n = 765)						
Quintile^c^						
First	1 [Reference]	NA		1 [Reference]	NA	
Second	0.89 (0.74-1.09)	.26	<.001	1.03 (0.83-1.29)	.77	.07
Third	0.62 (0.48-0.80)	<.001		0.84 (0.61-1.13)	.25	
Fourth	0.86 (0.67-1.09)	.21		1.20 (0.89-1.61)	.23	
Fifth	0.61 (0.47-0.79)	<.001		0.81 (0.58-1.11)	.19	
Stillbirth (n = 38)						
Quintile^c^						
First	1 [Reference]	NA		1 [Reference]	NA	
Second	0.85 (0.39-1.87)	.68	.34	0.96 (0.40-2.29)	.92	.57
Third	0.12 (0.01-0.63)	.05		0.18 (0.01-1.00)	.11	
Fourth	0.65 (0.21-1.77)	.42		0.79 (0.20-2.65)	.71	
Fifth	0.71 (0.25-1.82)	.49		1.08 (0.32-3.49)	.90	

Abbreviations: aMed, Alternate Mediterranean Diet; APO, adverse pregnancy outcome; NA, not applicable; OR, odds ratio.

^a^ Logistic regression models were used to evaluate associations of aMed score quintiles with odds of having any and individual APOs.

^b^ Model is adjusted for age, educational level, race and ethnicity, marital status, obesity, smoking, and family history of cardiovascular disease.

^c^ aMed score quintiles: first, aMed score 0-2; second, aMed score 3-4; third, aMed score 5; fourth, aMed score 6; and fifth, aMed score 7-9.

### Associations of aMed Score Components With APOs

When evaluating aMed score components in association with the primary outcome, plant-based foods were inversely associated with APOs. Specifically, adherence to the vegetable, fruit, and legume metrics was associated with lower odds of developing any APO (vegetables: aOR, 0.83 [95% CI, 0.74-0.93]; fruits: aOR, 0.89 [95% CI, 0.80-1.00]; and legumes: aOR, 0.77 [95% CI, 0.69-0.86]; Figure). Concordance with the fish guideline was also associated with lower odds of developing any APO (aOR, 0.86 [95% CI, 0.77-0.96]). The nut, whole grain, fat, red meat, and alcohol component scores were not significantly associated with risk of developing any APO. In analyses evaluating the associations of aMed score components with individual APOs, differential associations were observed (eTables 2 and 3 in Supplement 1). For preeclampsia or eclampsia, higher intakes of vegetables, fruits, and fish were associated with lower risk, while higher intakes of vegetables and lower intakes of red and processed meat were associated with lower odds of developing gestational diabetes.

**Figure.**
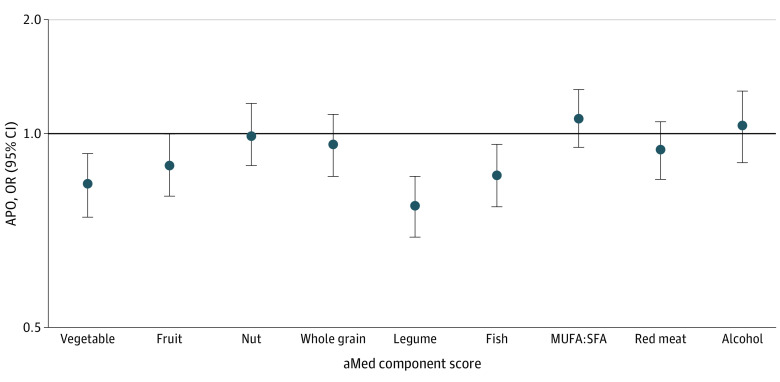
Multivariable-Adjusted Associations of the Alternative Mediterranean Diet (aMed) Score Components With Odds of Developing Any Adverse Pregnancy Outcome (APO) MUFA indicates monounsaturated fatty acids; SFA, saturated fatty acids.

### Subgroup Analyses

There was no significant interaction in the association between aMed score and any APO by prepregnancy BMI category, race, or ethnicity. However, we did observe a significant interaction by maternal age group. In stratified analyses, while protective associations were detected in both groups, the associations were stronger in women with advanced maternal age (aOR, 0.52 [95% CI, 0.33-0.81] for those aged ≥35 years; *P* = .004; vs aOR, 0.85 [95% CI, 0.73-0.99] for those aged <35 years; *P* = .04).

## Discussion

In this prospective cohort study of geographically and racially and ethnically diverse nulliparous US women, greater adherence to a Mediterranean diet pattern around the time of conception was associated with lower odds of developing any APO, particularly preeclampsia or eclampsia and gestational diabetes. We detected a dose-response association, highlighting that women with the highest concordance to this diet pattern prior to conception had the lowest risk of developing APOs. Different aspects of the Mediterranean diet were associated with individual APOs, but generally higher intakes of vegetables, fruits, legumes, fish, and whole grains and lower intakes of red and processed meat were associated with lower risk of APOs. Taken together, our findings demonstrate that in US women, adoption of a Mediterranean diet pattern may represent an important lifestyle approach for the prevention of APOs, particularly in women with advanced maternal age among whom risk for APOs is elevated.^2^ There were no significant differences in associations of the aMed score with APOs by race, ethnicity, or prepregnancy BMI, suggesting that there may be a benefit associated with this diet pattern for women of all racial and ethnic backgrounds, with and without obesity.

Adherence to the Mediterranean diet pattern in this cohort, as captured by the mean aMed score, was similar to prior studies of US women, with most participants in the low and moderate categories.^7,30^ Our findings are also consistent with the few prior observational studies demonstrating that more favorable diet quality around the time of conception and throughout pregnancy is associated with lower risk of APOs.^5,13,14,15,31^ However, only 3 of these studies evaluated an aMed diet pattern in association with APOs.^13,14,15^ In 1 study of 1076 women from 10 Mediterranean countries, a Mediterranean pattern of eating was associated with lower incidence of gestational diabetes (8% vs 12% when comparing the highest vs lowest tertiles).^13^ A US study evaluated the association of an aMed score with risk of any APO (defined as gestational diabetes, gestational hypertension, preeclampsia, and preterm birth) in 1887 pregnant women.^14^ In that study, those in the highest vs lowest quartiles of an aMed score, based on diet data collected at 8 to 13 weeks’ gestational age, had approximately 50% lower risk of developing any pregnancy complication (*P* = .001 for trend). However, when associations with individual APOs were evaluated, higher aMed scores tended to be associated with lower risk, but none of these results were statistically significant. That study used a different definition for the composite outcome and acknowledged that their sample size and modest number of APOs may have limited the statistical power to detect associations with individual APOs.

Interventional studies in European women have evaluated the association of a Mediterranean-style diet with risk of developing gestational diabetes and demonstrated a protective association, consistent with our findings in the present study.^32,33^ In a Spanish study of 874 pregnant women at 8 to 12 weeks’ gestational age, the intervention group had 25% lower risk of developing gestational diabetes compared with the control group.^33^ Similarly, in the ESTEEM (Effect of Simple, Targeted Diet in Pregnant Women With Metabolic Risk Factors on Pregnancy Outcomes) trial, British women randomized to receive dietary counseling based on a Mediterranean-style diet vs usual care had a 35% reduction in odds for developing gestational diabetes.^32^ Finally, in the IMPACT BCN (Improving Mothers for a Better Prenatal Care Trial Barcelona) trial, a structured Mediterranean diet intervention reduced the risk of having a small-for-gestational-age infant in high-risk Spanish women.^34^ Although the aMed score was associated with overall APO risk in our study, we did not observe an association with having a small-for-gestational-age infant. This discrepancy could be owing to differences in study sample characteristics or the low incidence of this outcome and a lack of power.

The observed associations between the aMed score and developing preeclampsia or eclampsia and gestational diabetes are biologically plausible, as adherence to a Mediterranean diet pattern has been linked to decreased adiposity; favorable glycemic profiles; lower systolic and diastolic blood pressure, inflammation, and insulin resistance; and better endothelial function.^35,36,37^ These factors have all been implicated in the causes of preeclampsia and gestational diabetes.^38,39,40,41^ It is possible that the significant association found for preeclampsia but not gestational hypertension is due to the association of the Mediterranean diet pattern with antiangiogenic, inflammatory, and immune-modulated pathways underlying the development of preeclampsia. Alternatively, it is possible that the aMed score does not adequately capture the aspects of diet associated with risk for gestational hypertension.

Our result that multiple aMed score components are associated with odds of developing APOs is consistent with the literature demonstrating that dietary patterns before and/or during pregnancy characterized by higher intakes of plant-based foods and fish and lower intakes of red and processed meat are associated with lower risks of multiple APOs, although much of this research has been conducted in healthy, non-Hispanic White women.^5,42,43^

### Strengths and Limitations

Our study has notable strengths, including the geographic, racial, and ethnic diversity that is representative of the US population; the rigorous assessment of maternal sociodemographic, lifestyle, and clinical characteristics including adjudicated APOs in the nuMoM2b cohort; and the prospective study design with diet data collected prior to occurrence of APOs, which enables the establishment of temporality and minimizes risk for reverse causality. The use of a validated, detailed, and widely used FFQ to measure habitual diet and the assessment of diet quantity and quality shortly after the time of interest enhances the quality and fidelity of dietary recall. In addition, the use of an aMed score, representing a recommended a priori–defined healthy dietary pattern adapted for US populations and previously linked to several adverse health outcomes, is another strength of our study, as results could inform dietary strategies to improve health during pregnancy.

There are several limitations worth noting. First, self-reported diet is prone to measurement error, and misclassification and recall bias likely attenuated associations toward the null given the prospective study design. Second, participants in the nuMoM2b cohort had access to prenatal care at a large academic medical center during their first trimester of pregnancy; this factor likely also resulted in underestimation of the associations (bias toward the null). Third, because our study is observational, we are not able to establish causality. Fourth, we had limited power to conduct subgroup analyses. Fifth, we cannot rule out the possibility of residual confounding by unknown or unmeasured factors, particularly associated with socioeconomic position and neighborhood characteristics. For example, we did not have data on the area deprivation index or living in a food desert, which could influence the association between diet and APOs.

## Conclusions

To our knowledge, our study represents the largest population-based US prospective cohort study examining a Mediterranean diet pattern around the time of conception and its association with odds of developing any APO and individual APOs and is the first to evaluate potential differences in these associations by maternal age, BMI, race, and ethnicity. We demonstrate that a Mediterranean diet pattern is associated with lower risk of developing any APO and multiple individual APOs in US women, with evidence of a dose-response association. Our findings add to the growing body of evidence demonstrating that the Mediterranean diet pattern may play an important role in preserving the health of women across the lifespan, including during pregnancy.^10,12,13,14^ Long-term intervention studies are needed to assess whether promoting a Mediterranean-style diet pattern around the time of conception and throughout pregnancy can prevent APOs or reduce their downstream associations with future CVD risk. This may be particularly useful to study in pregnant persons at high risk for APOs.

## Notes

Supplement 1. **eTable 1.** Prevalence of Adverse Pregnancy Outcomes (APOs) Stratified by Alternate Mediterranean Diet (aMed) Dietary Compliance **eTable 2.** Univariable and Multivariable Adjusted Results for Alternate Mediterranean Diet (aMed) Score Components With Preclampsia/Eclampsia **eTable 3.** Univariable and Multivariable Adjusted Results for Alternate Mediterranean Diet (aMed) Score Components With Gestational Diabetes **eFigure.** Analysis Sample Selection Flow Diagram

Supplement 2.
Data Sharing Statement
