# Supplementary material for: Association of a Mediterranean Diet Pattern With Adverse Pregnancy Outcomes Among US Women
Source: JAMA Netw Open. 2022 Dec 22;5(12):e2248165. doi: 10.1001/jamanetworkopen.2022.48165 (PMC9857221; doi:10.1001/jamanetworkopen.2022.48165)
Supplement: Supplement 1. — eTable 1. Prevalence of Adverse Pregnancy Outcomes (APOs) Stratified by Alternate Mediterranean Diet (aMed) Dietary Compliance eTable 2. Univariable and Multivariable Adjusted Results for Alternate Mediterranean Diet (aMed) Score Components With Preclampsia/Eclampsia eTable 3. Univariable and Multivariable Adjusted Results for Alternate Mediterranean Diet (aMed) Score Components With Gestational Diabetes eFigure. Analysis Sample Selection Flow Diagram [file jamanetwopen-e2248165-s001.pdf]

Supplementary Online Content

Makarem N, Chau K, Miller EC, et al. Association of a Mediterranean diet pattern with adverse pregnancy outcomes among US women. *JAMA Netw Open.* 2022;5(12):e2248165. doi:10.1001/jamanetworkopen.2022.48165

eTable 1. Prevalence of Adverse Pregnancy Outcomes (APOs) Stratified by Alternate Mediterranean Diet (aMed) Dietary Compliance
eTable 2. Univariable and Multivariable Adjusted Results for Alternate Mediterranean Diet (aMed) Score Components With Preclampsia/Eclampsia
eTable 3. Univariable and Multivariable Adjusted Results for Alternate Mediterranean Diet (aMed) Score Components With Gestational Diabetes
eFigure. Analysis Sample Selection Flow Diagram

This supplementary material has been provided by the authors to give readers additional information about their work.

eTable 1. Prevalence of Adverse Pregnancy Outcomes (APOs) Stratified by Alternate Mediterranean Diet (aMed) Dietary Compliance

	Low (N=2980)	Moderate (N=2430)	High (N=2388)	p-value*
**Any APO (N=2747)**	1137 (38.2%)	849 (34.9%)	761 (31.9%)	<0.001
**Preeclampsia/eclampsia (N=606)**	276 (9.3%)	184 (7.6%)	146 (6.1%)	<0.001
**Gestational hypertension (N=1106)**	443 (14.9%)	334 (13.7%)	329 (13.8%)	0.419
**Gestational diabetes (N=300)**	122 (4.1%)	104 (4.3%)	74 (3.1%)	0.066
**Preterm birth (N=544)**	229 (7.7%)	167 (6.9%)	148 (6.2%)	0.102
**Small for gestational age (N=765)**	331 (11.1%)	225 (9.3%)	209 (8.8%)	0.010
**Stillbirth (N=38)**	19 (0.6%)	8 (0.3%)	11 (0.5%)	0.262

aMed score categories defined as low (0-3), moderate (4-5), high (6-9) compliance

© 2022 Makarem N et al. *JAMA Network Open.*

eTable 2. Univariable and Multivariable Adjusted Results for Alternate Mediterranean Diet (aMed) Score Components With Preclampsia/Eclampsia

**Outcome**	**Unadjusted**		**Adjusted***	
	**OR (95% CI)**	**p-value**	**OR (95% CI)**	**p-value**
Vegetable Score	0.73 (0.62,0.87)	<0.001	0.78 (0.63,0.95)	0.01
Fruit Score	0.70 (0.59,0.83)	<0.001	0.73 (0.60,0.89)	0.002
Nut Score	0.90 (0.76,1.07)	0.23	0.98 (0.81,1.19)	0.82
Whole Grain Score	0.77 (0.65,0.91)	0.002	0.86 (0.71,1.04)	0.13
Legumes Score	0.79 (0.67,0.94)	0.01	0.91 (0.75,1.10)	0.32
Fish Score	0.78 (0.66,0.92)	0.003	0.83 (0.69,1.00)	0.05
MUFA:SFA Score	0.95 (0.81,1.13)	0.58	0.94 (0.78,1.13)	0.52
Red Meat Score	0.86 (0.73,1.02)	0.08	0.96 (0.80,1.15)	0.67
Alcohol Score	0.85 (0.69,1.04)	0.12	0.91 (0.72,1.14)	0.42

* Adjusting for age, education, race/ethnicity, marital status, body mass index, smoking, family history of cardiovascular disease.

MUFA : SFA = monounsaturated to saturated fat ratio

© 2022 Makarem N et al. *JAMA Network Open.*

eTable 3. Univariable and Multivariable Adjusted Results for Alternate Mediterranean Diet (aMed) Score Components With Gestational Diabetes

**Outcome**	**Unadjusted**		**Adjusted***	
	**OR (95% CI)**	**p-value**	**OR (95% CI)**	**p-value**
Vegetable Score	0.88 (0.70,1.11)	0.30	0.72 (0.55,0.95)	0.02
Fruit Score	0.86 (0.68,1.08)	0.20	0.84 (0.64,1.10)	0.21
Nut Score	0.97 (0.77,1.22)	0.77	0.94 (0.73,1.23)	0.67
Whole Grain Score	0.82 (0.65,1.03)	0.09	0.83 (0.64,1.08)	0.17
Legumes Score	0.95 (0.76,1.20)	0.69	0.99 (0.76,1.29)	0.94
Fish Score	0.94 (0.75,1.19)	0.61	0.84 (0.65,1.09)	0.19
MUFA:SFA Score	1.00 (0.79,1.26)	0.99	0.95 (0.73,1.24)	0.71
Red Meat Score	0.69 (0.55,0.87)	0.002	0.73 (0.57,0.95)	0.02
Alcohol Score	0.94 (0.70,1.24)	0.66	0.92 (0.67,1.24)	0.58

* Adjusting for age, education, race/ethnicity, marital status, body mass index, smoking, family history of cardiovascular disease.

MUFA : SFA = monounsaturated to saturated fat ratio

© 2022 Makarem N et al. *JAMA Network Open.*

eFigure. Analysis Sample Selection Flow Diagram

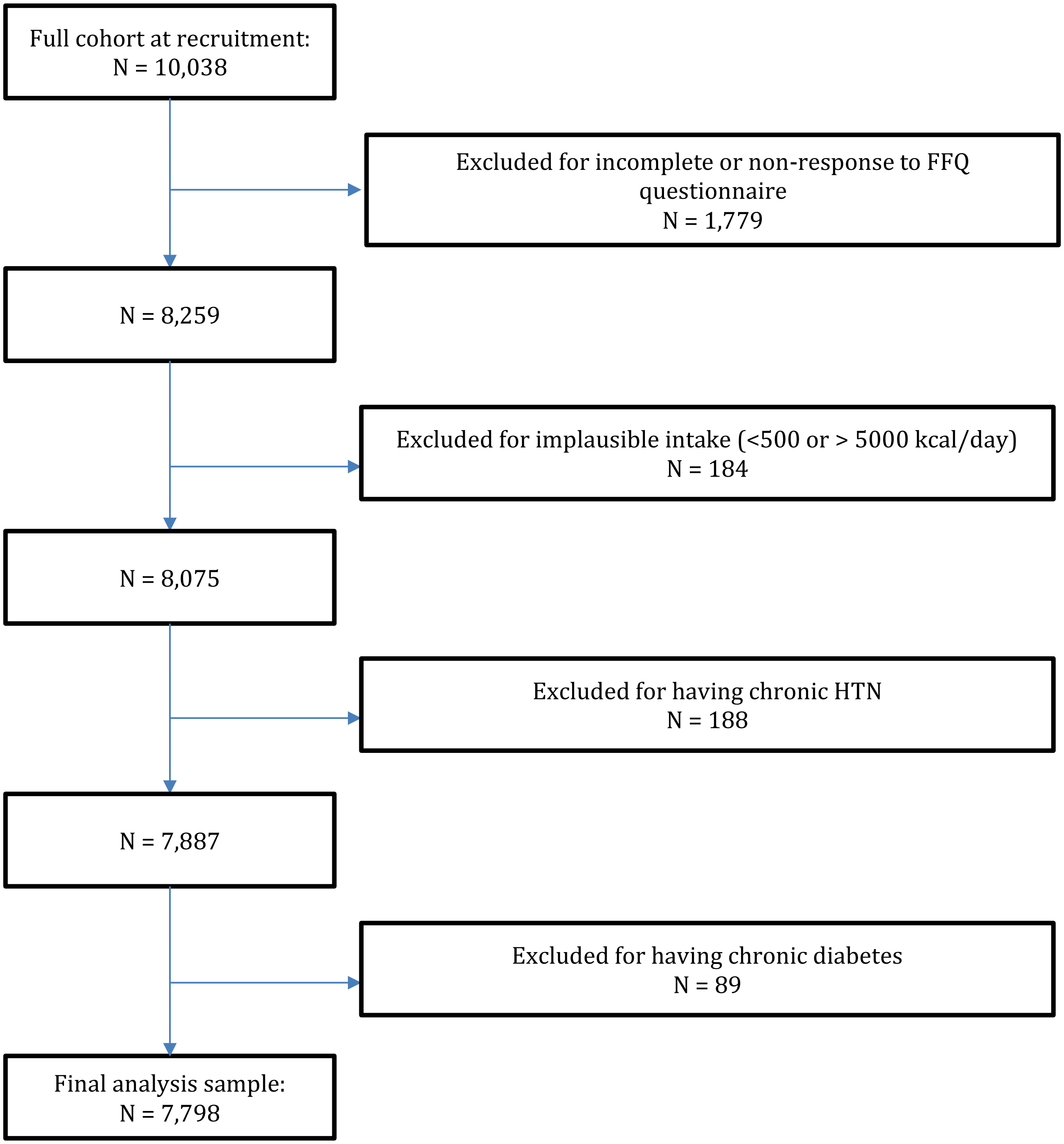

© 2022 Makarem N et al. *JAMA Network Open*.
