# Supplementary material for: Association of a Mediterranean Diet Pattern With Adverse Pregnancy Outcomes Among US Women
Source: JAMA Netw Open. 2022 Dec 22;5(12):e2248165. doi: 10.1001/jamanetworkopen.2022.48165 (PMC9857221; doi:10.1001/jamanetworkopen.2022.48165)
Supplement: Supplement 2. — Data Sharing Statement [file jamanetwopen-e2248165-s002.pdf]

Data Sharing Statement

Makarem. Association of a Mediterranean Diet Pattern With Adverse Pregnancy Outcomes Among US Women. *JAMA Netw Open*. Published December 22, 2022. doi:10.1001/jamanetworkopen.2022.48165

Data
Data available: Yes
Data types: Deidentified participant data
How to access data: Deidentified patient data is available on DASH https://dash.nichd.nih.gov/study/226675
When available: beginning date: 10-14-2022

Supporting Documents
Document types: None

Additional Information
Who can access the data: Please follow the instructions on the DASH website to access data. https://dash.nichd.nih.gov/study/226675
Types of analyses: Please follow the instructions on the DASH website to access data. https://dash.nichd.nih.gov/study/226675
Mechanisms of data availability: Please follow the instructions on the DASH website to access data. https://dash.nichd.nih.gov/study/226675
Any additional restrictions: Please follow the instructions on the DASH website to access data. https://dash.nichd.nih.gov/study/226675
